# Supplementary material for: Selection of tRNA Genes in Human Breast Tumours Varies Substantially between Individuals
Source: Cancers (Basel). 2023 Jul 12;15(14):3576. doi: 10.3390/cancers15143576 (PMC10377016; doi:10.3390/cancers15143576)
Supplement: Supplementary file 1 [file cancers-15-03576-s001.zip › cancers-2476438-supplementary.pdf]

## Supplementary figures

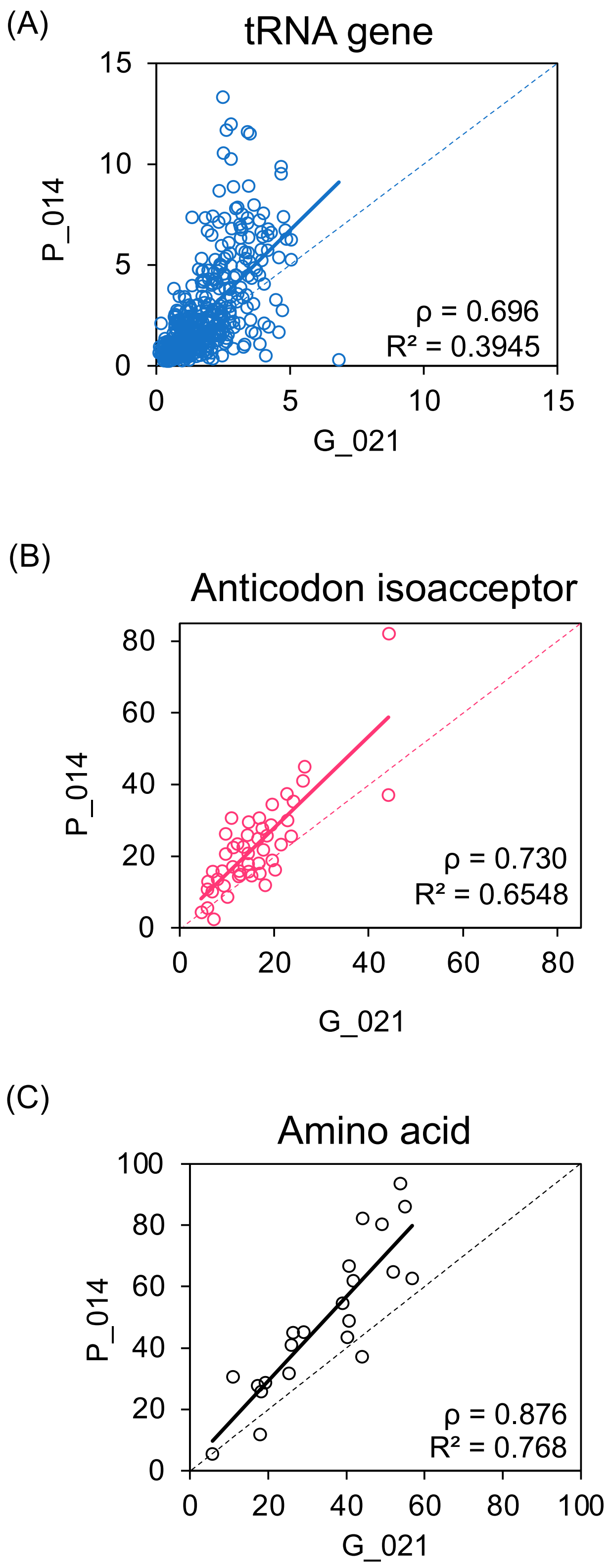


**Supplementary Figure S1.** Variation between cancers P_014 and G_021 of H3K27ac enrichment at tRNA genes and families. (**A-C**) Plots comparing H3K27ac Q-values at 428 tRNA gene loci (**A**), 48 anticodon isoacceptor families (**B**), and 21 amino acid isotype families (**C**) in primary tumours P_014 and G_021, which had overall relative H3K27ac enrichment across the 40 tumours and 428 tRNA genes of 1076 and 754, respectively. Spearman's rank correlation coefficient is shown (ρ).

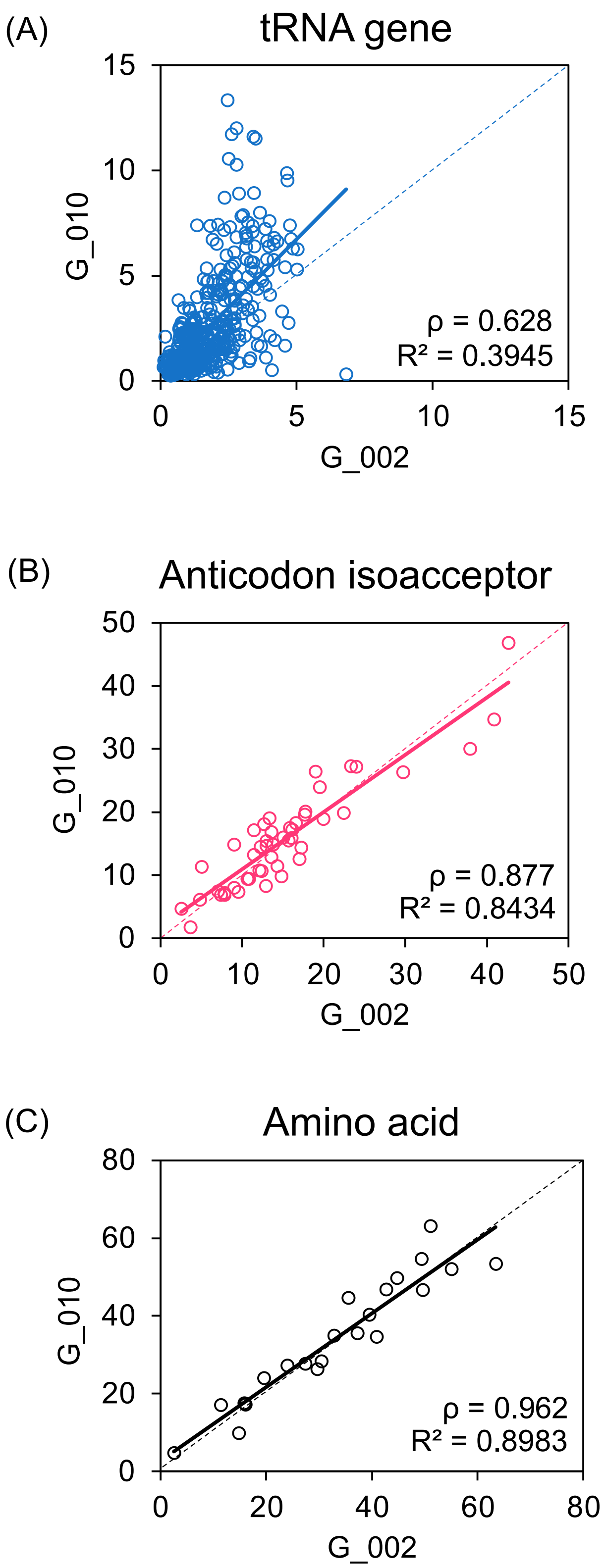


**Supplementary Figure S2.** Variation between cancers G_002 and G_010 of H3K27ac enrichment at tRNA genes and families. (**A-C**) Plots comparing H3K27ac Q-values at 428 tRNA gene loci (**A**), 48 anticodon isoacceptor families (**B**), and 21 amino acid isotype families (**C**) in primary tumours G_010 and G_002, which were chosen because they share very similar overall H3K27ac enrichment across the 40 tumours and 428 tRNA genes (756 and 619, respectively). Spearman's rank correlation coefficient is shown (ρ).

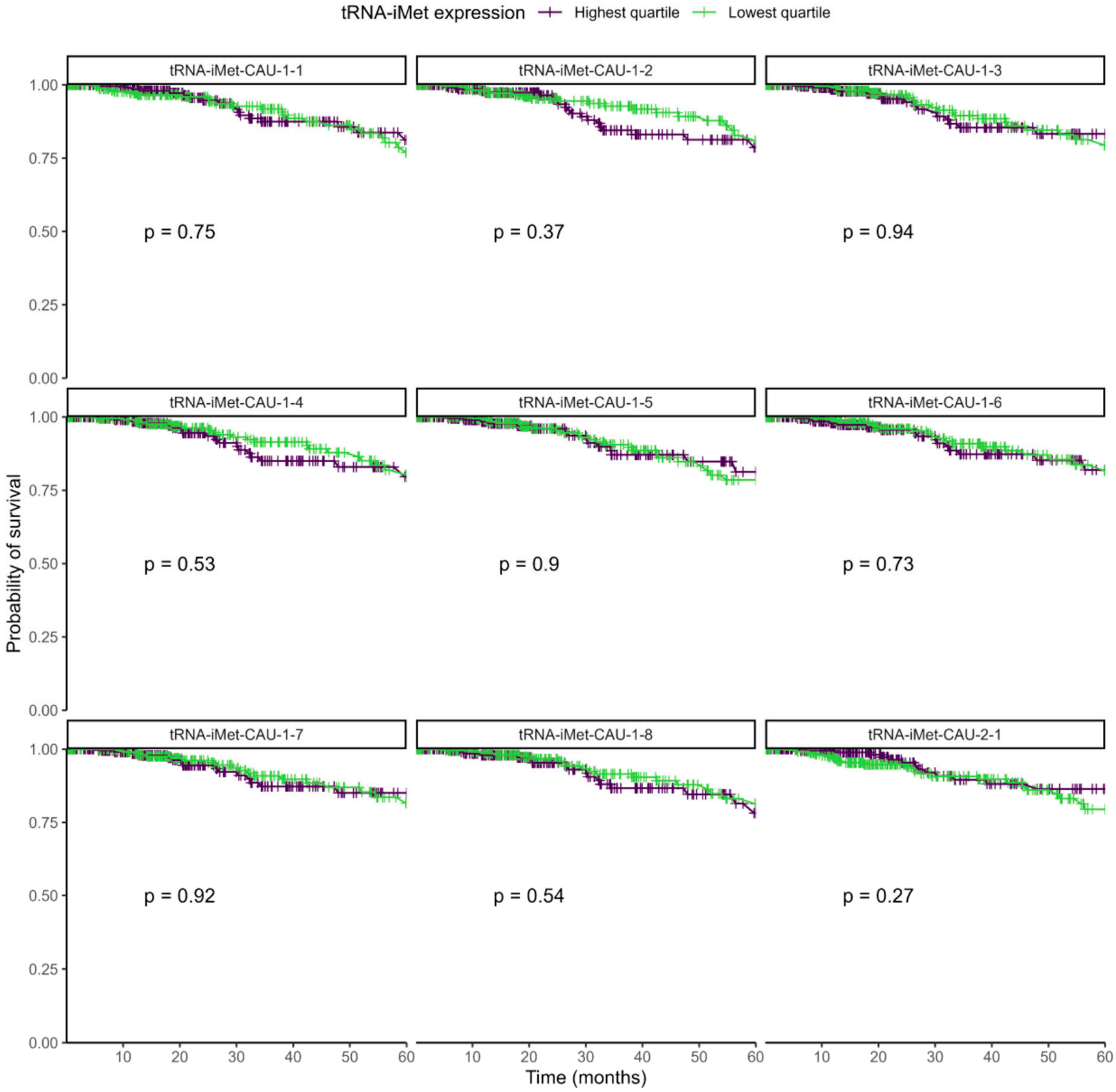


**Supplementary Figure S3.** Survival of patients with breast invasive carcinomas expressing upper quartile (purple) or lower quartile (green) levels of each individual tRNA-iMet-CAU.
